# Quanfima: An open source Python package for automated fiber analysis of biomaterials

**DOI:** 10.1371/journal.pone.0215137

**Published:** 2019-04-11

**Authors:** Roman Shkarin, Andrei Shkarin, Svetlana Shkarina, Angelica Cecilia, Roman A. Surmenev, Maria A. Surmeneva, Venera Weinhardt, Tilo Baumbach, Ralf Mikut

**Affiliations:** 1 Laboratory for Applications of Synchrotron Radiation, Karlsruhe Institute of Technology, Karlsruhe, Germany; 2 Institute for Automation and Applied Computer Science, Karlsruhe Institute of Technology, Karlsruhe, Germany; 3 Physical Materials Science and Composite Materials Centre, Research School of Chemistry & Applied Biomedical Sciences, National Research Tomsk Polytechnic University, Tomsk, Russia; 4 Institute for Photon Science and Synchrotron Radiation, Karlsruhe Institute of Technology, Eggenstein-Leopoldshafen, Germany; 5 Centre for Organismal Studies, COS, Heidelberg University, Heidelberg, Germany; Universidade de Lisboa, PORTUGAL

## Abstract

Hybrid 3D scaffolds composed of different biomaterials with fibrous structure or enriched with different inclusions (i.e., nano- and microparticles) have already demonstrated their positive effect on cell integration and regeneration. The analysis of fibers in hybrid biomaterials, especially in a 3D space is often difficult due to their various diameters (from micro to nanoscale) and compositions. Though biomaterials processing workflows are implemented, there are no software tools for fiber analysis that can be easily integrated into such workflows. Due to the demand for reproducible science with Jupyter notebooks and the broad use of the Python programming language, we have developed the new Python package quanfima offering a complete analysis of hybrid biomaterials, that include the determination of fiber orientation, fiber and/or particle diameter and porosity. Here, we evaluate the provided tensor-based approach on a range of generated datasets under various noise conditions. Also, we show its application to the X-ray tomography datasets of polycaprolactone fibrous scaffolds pure and containing silicate-substituted hydroxyapatite microparticles, hydrogels enriched with bioglass contained strontium and alpha-tricalcium phosphate microparticles for bone tissue engineering and porous cryogel 3D scaffold for pancreatic cell culturing. The results obtained with the help of the developed package demonstrated high accuracy and performance of orientation, fibers and microparticles diameter and porosity analysis.

## Introduction

Biomaterials are often designed to mimic chemical and physical properties, for instance shape, of biological systems [1–3]. The development of special fibrous and porous three-dimensional (3D) structures, so-called scaffolds, has gained popularity in the field of tissue engineering (TE) [4–6]. Such structures can replace and treat damaged body tissues[5]. The detailed analysis of the fibrous structure is essential to reveal dependencies between biomaterial properties and its performance in a tissue. For instance, controlling the fiber orientation in the scaffolds fabrication process allows for advanced customized solutions that promote faster and higher-quality treatment in many fields of TE. For bones, TE requires scaffolds with both, randomly oriented and aligned structures to mimic a native extracellular matrix (ECM) and to ensure appropriate mechanical properties [7]. In contrast, scaffolds designed for nerve and blood vessels are aimed to recreate the natural architecture of tissues with aligned fibers as closely as possible [8,9]. Such property as the fiber diameter influences cell adhesion and growth kinetics [10–12]. Moreover, some scaffolds consist of bioactive particles with different size, that influence the porosity and efficiency properties of the matrix. The porosity of biomaterials is linked to the success of tissue ingrowth [13–15]. The development of biomaterials with desired properties requires 3D characterization of their structure with a precise, ideally automatic, computational analysis.

There are a number of imaging techniques to characterize biomaterials [16]. Scanning electron microscopy allows to image of the biomaterial surface with high resolution and to study its morphological properties and composition. Atomic force microscopy is a precise tool for measuring the topography of the sample surface. Confocal laser scanning microscopy enables to perform a 3D characterization of the sample since it can produce high-resolution optical images at different depth levels. Despite of its relatively small penetration depth, it has become a widely established method. Micro-computed tomography (micro-CT) is an X-ray imaging technique that allows investigate the density and microarchitecture of mineralized tissues (e.g., bones, teeth) and soft tissues and biomaterials prepared in a specific way. This approach produces a series of radiographic images of the sample from different views, which subsequently can be reconstructed to reveal 3D information about the sample up to a micrometer resolution. All considered techniques produce datasets presented as an image or a sequence of images describing the investigated biomaterials. So far, these datasets must be processed using tailored image analysis methods to obtain a quantitative characterization of the biomaterial microarchitecture.

Over the past decades, several approaches for fiber orientation analysis of two-dimensional (2D) datasets have been proposed: line detection based on the Hough transform for the analysis of collagen fibers [17]; the computation of an intensity gradient at every pixel position to quantify the orientation of cytoskeletal [18] and collagen fibers [19]; Fourier analysis of spatial frequency components to determine the orientation of nanofibrous and nonwoven layers of textile materials [20,21], fibers in electrospun materials [22–24], collagen fibers, and fibroblast proliferation [25]. Although, these methods were applied successfully for the analysis of various biomaterials, the datasets were 2D. Only a few methods allow the quantification of fiber orientation in 3D datasets, for example to analyze reinforced composites [26–31]. Some approaches were based on detecting elliptical footprints on virtual 2D sections [32–35] or a vector summation algorithm [36–38].

Presently, there are various proprietary and open-source solutions available to conduct fiber analysis in 2D or 3D. Among the proprietary software, FEI Avizo 3D with Fiber Analysis module, the Automated Fiber Analysis included in the Software shipped with Phenom Scanning Electron Microscopes (SEM), and VGStudio MAX with its Fiber Composite Material Analysis Module, are commonly used. In addition, a vast variety of open-source solutions is available, most noticeable plug-ins for ImageJ [39] and the stand-alone application FiberScout [40] which is mainly aimed at the 3D analysis of fiber-reinforced polymers for industrial computed tomography (CT). The plug-ins for ImageJ include DiameterJ [41], FibrilTool [42], and OrientationJ [43] which offer the analysis of 2D images acquired with various imaging techniques like light and electron microscopy, micro-CT, and others.

Even though the mentioned software serves as good hands-on tools for researchers, none of them can process both 3D and 2D data equally well. Most plugins are oriented only to a single aspect of fiber characterization such that a complete analysis can be performed only with multiple software tools. Moreover, the lack of integration capabilities between these plugins impedes implementation of automated workflows and hence is time consuming. Given the extend of work that would be required to combine and link these algorithms in a complete workflow, we have developed a Python package for multi-dimensional full morphological analysis of fibrous structures in biological tissues and materials.

The Python programming language [44] became very popular for data analysis among researchers from various areas [45]. It has a steep learning curve for beginners because the user operates with high-level abstractions hiding all low-level mechanisms and hardware communications. Thus, one can concentrate on algorithm development rather than having to think, e.g., about the nuances of memory management. In the past few years, several Python packages simplifying interaction and visualization of multi-dimensional data have been released. Among them are NumPy [46], SciPy [47], scikit-image [48], scikit-learn [49], Pandas [50], Statsmodels [51], Matplotlib (MPL) [52], and others.

The release of Jupyter notebook (former IPython notebook) [53] has opened a novel way for reproducible science. The platform allows writing a code in web-based interactive notebooks, run it on special servers with a high-throughput connection to large data storages, and share it with collaboration partners. The URLs of these notebooks or the notebooks themselves can be attached to a paper as a supplementary material to fill the gap between the high-level algorithm description, its implementation, and further use by other researchers [54].

In this paper we present the newly developed quanfima ("Quantitative Analysis of Fibrous Materials") package, which allows seamless integration of fiber analysis into third-party software and data processing workflows where Python is supported. The package provides automatic analysis of the foremost parameters required for biomaterial characterization, such as porosity, fiber orientation and diameter, number of particles, and inclusions. It facilitates the 3D visualization of fibrous biomaterials by mapping geo-coordinates to a color scheme, and the visualization of the orientation in a region-wise (plotting orientation vectors in regions of interest distributed across the image) as well as a pixel-wise (coloring pixels according to orientation in a neighborhood) fashion. We also added the Statsmodels package [51] to provide an intuitive interface for statistical data analysis (p-values). To ease further use of the package, we added a Jupyter notebook such that the analysis presented here can be reproduced and extended further for any type of biomaterials characterization.

## Materials and methods

### Generated datasets

**Fig 1:**
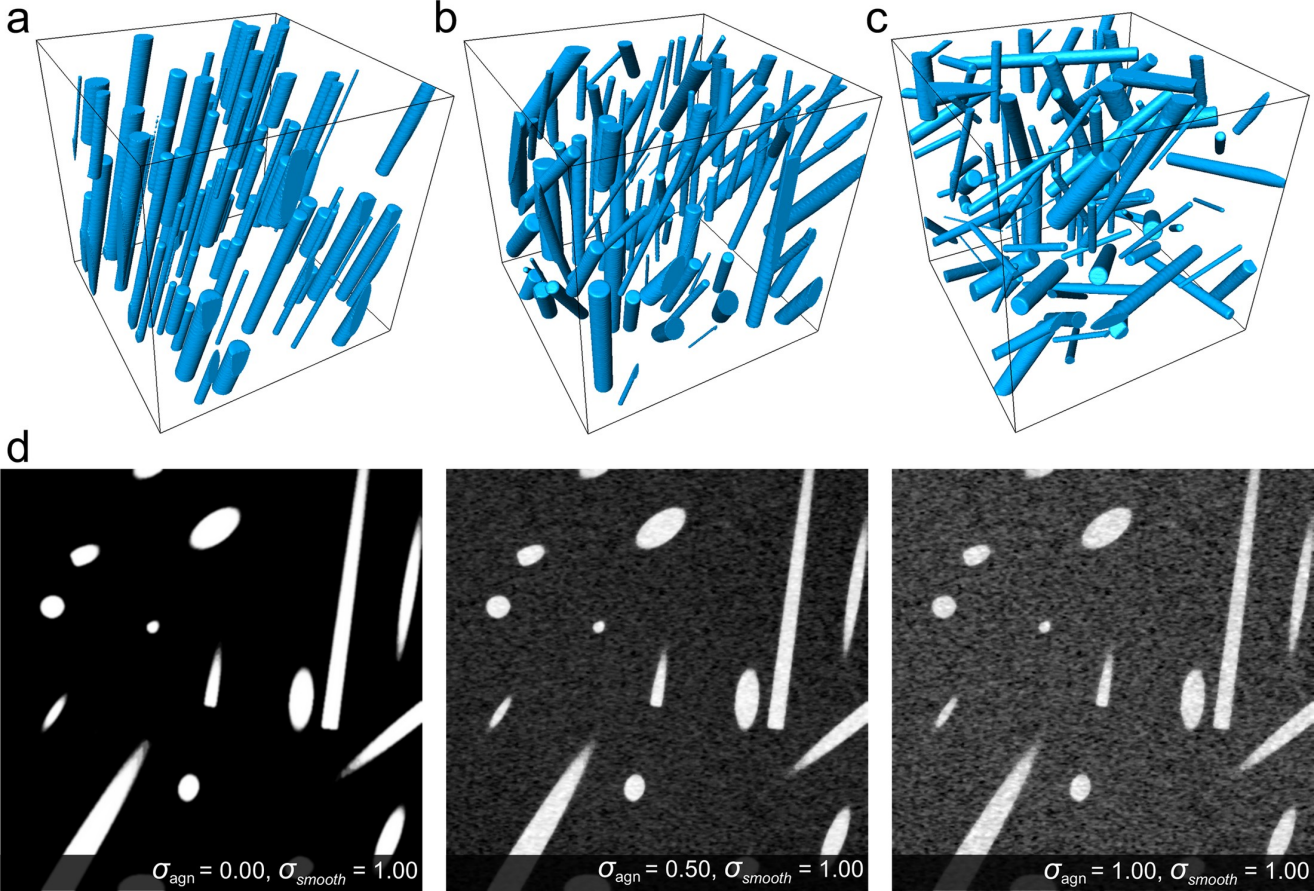
The generated datasets with different fiber configurations: a) aligned (A); b) moderately aligned (M); c) disordered (D); d) the central slice from the YZ-plane of generated disordered dataset contaminated with additive Gaussian noise and smeared with the Gaussian filter.

We have generated datasets with aligned, moderately aligned, and disordered fiber composition (Fig 1a). The size of each dataset is 512x512x512 voxels, the number of generated fibers ranges from 20 to 100. The fiber orientation of the aligned dataset is fixed at 27 and 15 degrees for azimuth and elevation angles, respectively. The moderately aligned orientation ranges from -45 to 45 degrees for the azimuth angle and from 0 to 45 degrees for elevation angle. In the case of the disordered dataset, the orientation diverges from -89 to 90 degrees for the azimuth angle and from 0 to 90 degrees for elevation angle. The diameter of each fiber ranges from 3 to 20 pixels, the fiber length is limited by range from 20% to 80% of the minimum side length of the volume. The separation between the fibers ranges from 3 to 10 pixels, and the threshold value of the number of coordinates located outside of the volume is 50%.

The generated datasets were contaminated with additive Gaussian noise and smeared with a Gaussian filter to determine the limitations of the orientation estimation algorithm. The standard deviation of noise σ_agn_ was 0.0, 0.5, 1.0, and 1.5, while for the smearing filter σ_smooth_ of 1.0 was used (Fig 1b).

### Experimental datasets

#### Pure PCL and PCL-SiHA composite scaffolds

For the fabrication of fibrous scaffolds polycaprolactone (PCL) and silicate-substituted hydroxyapatite (SiHA) biomaterials were chosen. The electrospinning process was employed in the fabrication of fibrous scaffolds with randomly oriented (rPCL) and well-aligned (wPCL) structures. The detailed description of biomaterials properties and electrospinning process settings are provided elsewhere [55]. The structure of the fabricated scaffolds was measured in a high-resolution micro-CT setup. The experiment was performed at the micro-imaging station placed at synchrotron radiation facility at the Karlsruhe Institute of Technology (KIT, Karlsruhe, Germany) [56]. The imaging setup used monochromatic beam in conjunction with an sCMOS Camera (sensor size 5.5 megapixels, 6.5 μm physical pixel size), a 200-μm-thick Lu3Al5O12 scintillator and a macroscope 3.6x from the Federal Institute for Materials Research and Testing, which allowed for a spatial resolution of approximately 1.8 μm with a field of view of 4.6×3.9 mm^2^. The samples were rotated with a step size of 0.24 degrees and exposed for 1 sec with 12 keV X-rays. The acquired data were reconstructed using the filtered backprojection (FBP) algorithm [57].

#### Hydrogels

The hydrogel samples consisted of bioglass enriched with strontium (BGSr) and alpha-tricalcium phosphate (α-TCP) were prepared according to the protocol described in [58,59]. Then, they were measured with a laboratory X-ray tube (Viscom X9160-D ED) at the Detector Lab of the Institute for Photon Science and Synchrotron Radiation of the Karlsruhe Institute of Technology (KIT, Karlsruhe, Germany). The electron beam energy and current were set to 60 keV and 120 μA, respectively. The signal was recorded with a Dexela 1207 CMOS camera with a physical pixel size of 74.8 μm together with a 150 μm thick CsI scintillator. The source to sample distance and source to detector distance were set to 3.5 and 70 cm, respectively, which resulted in a total magnification of 20x, an effective pixel size of 3.7 μm, and a field of view of 3.2 x 5.7 mm^2^. Each sample was rotated over 360 degrees with a step size of 0.3 degrees and exposed for 4 sec with X-rays. The collected projections were reconstructed with the filtered backprojection (FBP) algorithm adapted for the conical beam [60].

#### Cryogels

Cryogel scaffolds fabrication and cell culturing were done in the same way as in [61]. The tomography experiment was performed at the ID19 beamline of the European Synchrotron Radiation Facility (ESRF, Grenoble, France) using so-called "pink beam" radiation. The beam spectrum was filtered with 1.4 mm of Aluminium, which provided the resulting beam energy around 26 keV. The distance between the sample and the detector was set to 71 cm. The detector system was composed of a pco.edge camera and an optical system providing 4x magnification designed by Optique Peter, which resulted in an effective pixel size of 1.8 μm. 1500 projections were acquired for each sample with a step size of 0.12 degrees. Then, the collected datasets were processed with the so-called "quasi-particle" phase-retrieval algorithm [62] and reconstructed with the FBP algorithm [57].

### Benchmarking setup

The package validation was done at a workstation equipped with an Intel Xeon E5-2680 v2 processor, GeForce GTX TITAN 6GB graphical adapter and 256 GB of RAM.

## Results

The most frequently cited software tools for analysis of fibrous materials are compiled in Table 1, where functionality, easiness of software extensibility, and integration with other software by users without long programming experience are presented. FiberScout was developed for analysis of industrial CT datasets of fiber-reinforced materials [63]. It provides a wide range of features, which includes displaying a distribution of fiber lengths and orientation to clustering fibers with equivalent properties and selection of individual fibers by certain criteria. This tool is represented by a module for the open_iA platform, which is written in C++ and uses the ITK library [64]. Among the plug-ins for ImageJ, DiameterJ [41] was developed with a focus on the analysis of SEM micrograph images, providing information about orientation and diameter distributions. The OrientationJ [43] plug-in is aimed initially at the analysis of microscopy images and provides information about orientation, coherency, and energy within a specified ROI. Additionally, it can combine extracted data into a single image to enhance the visual perception. The FibrilTool [42] plug-in has been recently proposed for the study of fibrillar structures of microscopy images, extracting the information of average orientation along with an anisotropy value for a given region of interest (ROI). All these plug-ins are written in Java and run in a Java Virtual Machine, and cannot be easily integrated into the Python environment.

**Table 1:** Comparison of open source orientation analysis software with the proposed package.

FeatureName	FiberScout	DiameterJ	FibrilTool	OrientationJ	quanfima
**Language**	C++	Java	Java	Java	Python
**Integrability**	Not directly supposed	ImageJ macro language	ImageJ macro language	ImageJ macro language	Python environment
**Dimensionality**	3D	2D	2D	2D	3D and 2D
**Easiness of extensibility**	Hard	Medium	Medium	Medium	Easy
**Application focus**	CT	Microscopy	Microscopy	Microscopy	CT and Microscopy
**Local orientation estimation**	Yes	Yes	No	Yes	Yes
**Global orientation estimation**	Yes	Yes	Yes	Yes	Yes
**Diameter estimation**	Yes	Yes	No	No	Yes
**Fiber length estimation**	Yes	No	No	No	No
**Individual fiber selection**	Yes	No	No	No	No
**Fiber grouping**	Yes	No	No	No	No
**Chart plotting**	Yes	Yes	No	Yes	Yes
**Visualization**	Yes	Yes	Yes	Yes	Yes

In contrast to the mentioned software tools, our package is focused on parameters particularly required for biomaterials, such as fiber orientation and diameter, the distribution of inclusions, and porosity. Aside from processing, it provides a simple interface to visualize data and perform statistical analysis. The quanfima package offers both 2D and 3D analysis of data, whereas other solutions such as DiameterJ, OrientationJ, and FibrilTool are useful only for 2D analysis, and provide limited integration capabilities. Due to among the considered software only FiberScout provides capabilities for 3D analysis, we have compared the accuracy of orientation analysis of both software solutions on the generated dataset with the aligned fiber configuration. The results have shown, that azimuth and elevation angular errors with the window size of 64 pixels are smaller for quanfima (1.47±1.40, 0.58±0.62 degrees) in comparison to FiberScout (1.63±4.03, 0.31±0.89 degrees). It demonstrates that our implementation of the tensor-based approach can provide an accuracy comparable to the already existing implementation with a slight deviation of elevation angular error by 0.27 degree with respect to FiberScout. A direct comparison of both solutions is however impossible because of the difference in approaches to estimate orientation of fibers. FiberScout calculates orientation for every group of adjacent voxels forming a fiber, while quanfima estimates orientation for every voxel of a medial fiber axis, relying on its neighborhood. Thus, application of FiberScout is limited to non-intersecting fibers, which is typically not applicable to biomaterials, where fibers could be intertwined. Moreover, the approach for orientation estimation used in quanfima allows analyzing fibers of arbitrary configuration because orientation is estimated in a voxel-wise fashion. Also, as mentioned earlier, FiberScout is provided as a module for open_iA platform, and as well as ImageJ plug-ins require good programming skills and knowledge of inter-process communication to be integrable into Python-based data analysis workflows. In this respect, quanfima is useful for a broader community as it can be used as an external library in any projects created with the Python language (e.g., analysis workflows or applications).

## Quanfima architecture

**Fig 2:**
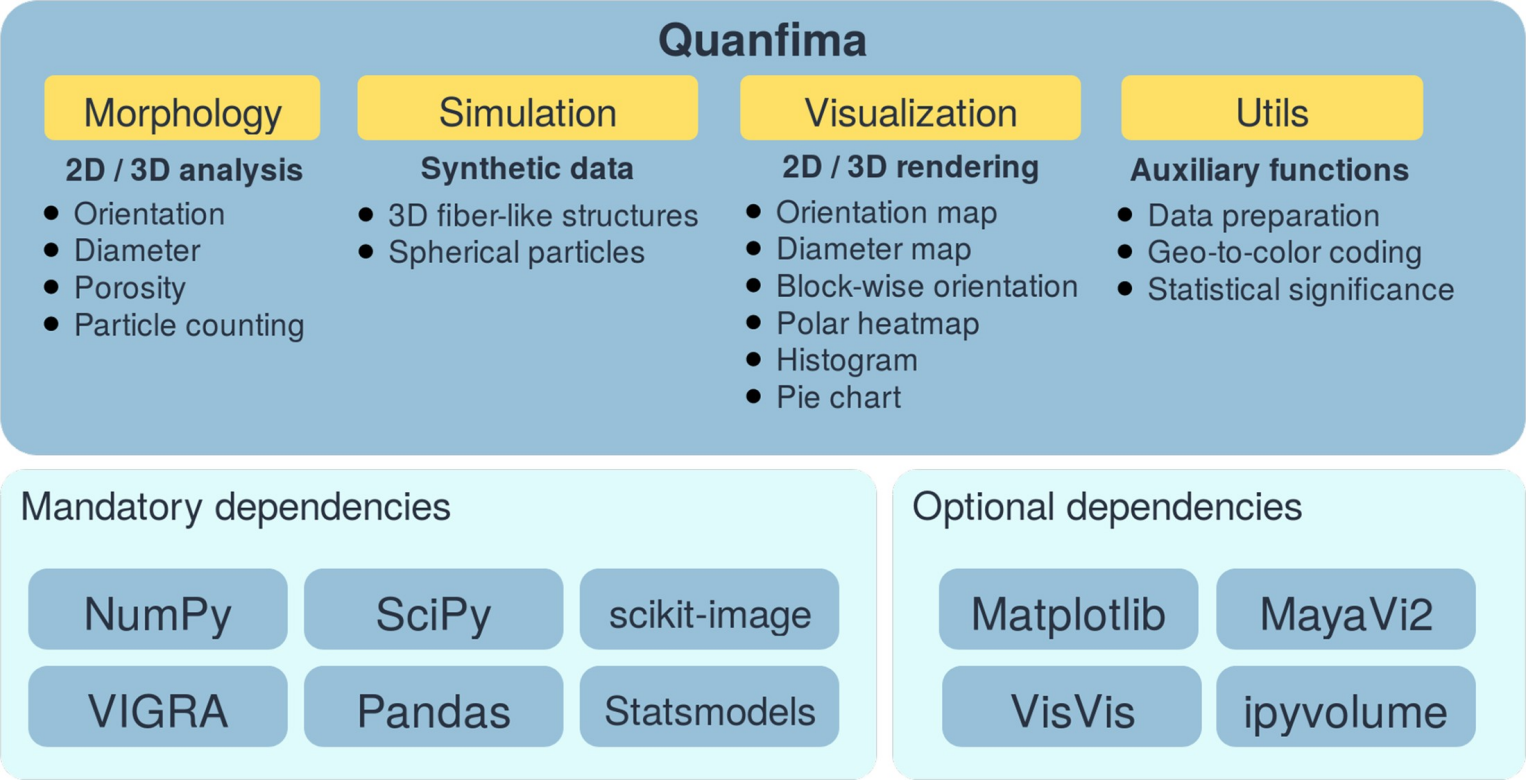
The general architecture of the quanfima package. Mandatory packages are included in a repository and serve as a base for quanfima. Optional packages are only necessary for the visualization module.

The quanfima package is based on several widespread third-party packages developed for data analysis, such as NumPy, SciPy, scikit-image, VIGRA, Pandas, and Statsmodels. We combined their capabilities to create a complete pipeline for characterization of biomaterials. The functionality of quanfima is separated into four modules (Fig 2).

The packages used by quanfima can be separated into mandatory and optional. The mandatory packages ensure a core functionality of analysis routines and are therefore essential for installation. The optional packages are required only by the visualization module and can be skipped if the user prefers to use other software for visualization. The dataset can be loaded as separate grayscale images, or as a stack of images representing a 3D volume.

In the following, we will describe the modules specifically developed by us to complete the package for analysis of biomaterials.

### Simulation

**Fig 3:**
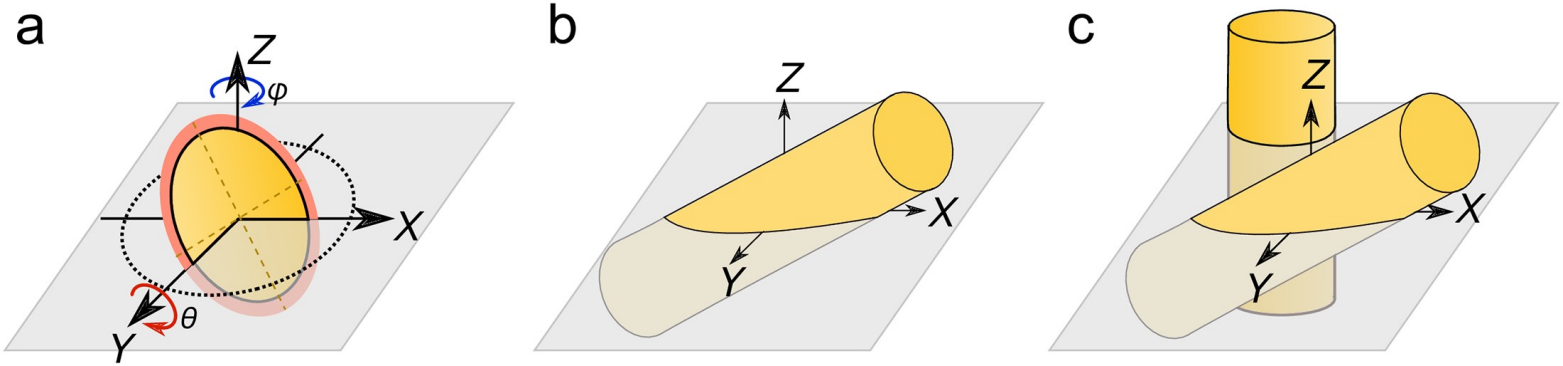
The synthetic fiber generation process: a) the cross-section rotation in a plane of the 3D volume, the orange region represents a gap for a given fiber; b) the cross-section propagation along the specified direction to form a fiber; c) adding the fiber into the 3D volume.

The primary module in the quanfima package allows the generation of synthetic datasets. Such datasets provide users with the ground-truth values which makes verification of imaging techniques or analysis algorithms possible. Here, we propose an algorithm for the generation of non-curved fiber-like structures in a 3D volume. The diameter and orientation of each fiber is represented by a diameter value, and azimuth and elevation angles are sampled from given ranges. It is assumed that individual fibers do not cross each other and are separated by a specified gap value. The length of the fibers is specified as a percentage with respect to the minimum side length of the volume. The fibers are added to the volume until a stop rule is satisfied. The stop rule can be a maximum number of fibers, the ratio between the volume of all fibers and the total volume, or the number of failed additions due to intersections of fibers. Each fiber is initiated as a set of points representing a circle of the specified diameter. Then these points are rotated by the sampled azimuth and elevation angles (Fig 3a) and propagated along the unit vector perpendicular to the profile (Fig 3b). Then, the obtained coordinates are transformed to array indices and used for checking on the placing conditions and forming a fiber in a three-dimensional array (Fig 3c).

To more closely simulate real fibers, the parameters of the fibers should be sampled from value ranges defined heuristically or analytically. If randomly oriented and tightly packed thin fibers in a small 3D volume are desired, the simulation process requires a high number of fibers, narrow distributions the gap value and the diameter and a broad distribution of orientation values. The simulated volume consists of two materials, where zero values represent the background and non-zero values the synthetic fibers. The produced dataset can be contaminated with an additive Gaussian noise of specified sigma to emulate the noise produced by a digital detector. The noise value is sampled from the noise distribution and applied to every point of the 3D volume independently. Since we measure physical dimensions in pixels, this allows us to control fiber parameters at the finest level and be versatile in terms of resolution. The maximal size of a simulated volume completely depends on the amount of RAM of the specific computer. An example of the generated datasets aligned, moderately aligned and disordered configurations are shown in Fig 1(a)–1(c). We generated datasets with a varied number of fibers from 20 to 100 in conditions of prohibited and allowed fiber intersection. The measured execution time shows a linear growth for fibers with intersections. For fibers with no intersections the execution time increases almost logarithmically with number of fibers (see S1a Fig). The increase of execution time is about factor of 3 times higher for not intersected fibers.

### Morphology

The module “Morphology” is the center of our package. It offers the analysis of generated and experimental fiber datasets, which includes the determination of diameter, orientation, and number of fibers, as well as allows porosity calculations.

#### Fiber orientation analysis

The fiber orientation analysis is performed on skeletonized binary data obtained via a segmentation procedure from the scikit-image package included in quanfima, or any other package which provides image segmentation capabilities.

**Fig 4:**
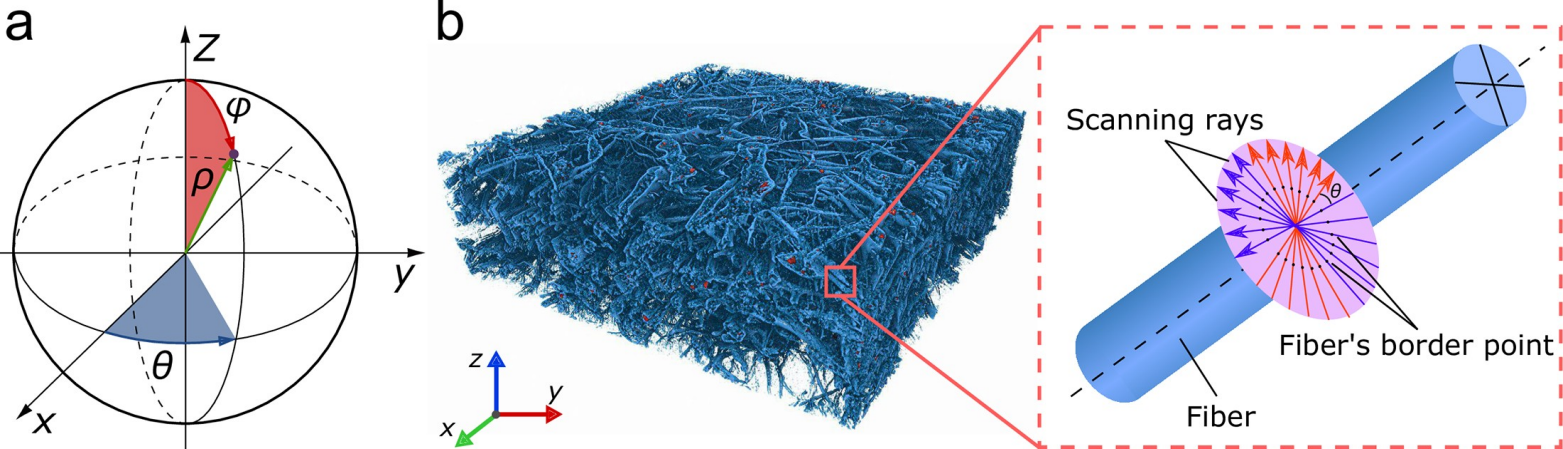
The representation of a fiber in a spherical coordinate system (a). 3D rendering of a rPCL scaffold with SiHA inclusions acquired with micro-CT with a schematic for the diameter estimation algorithm (b).

The fibers are characterized by their length ρ and their direction described by a unit vector (θ, φ) in a spherical coordinate system (Fig 4a). The azimuth angle θ specifies the orientation in the XY-plane, whereas φ is the elevation angle representing a tilt with respect to the Z-axis. To avoid angle mirroring, only unique angles are covered, i.e., angles vary from 0 to 90 degrees for elevation and from -90 to 90 degrees for azimuth only.

We implemented two methods for analysis of the fiber orientation, which are based on the Fourier spectrum [65] and the second-order structure tensor [27,30,66,67], respectively. In both cases, the user obtains a value or a pair of numbers representing the orientation angles in radians.

The first method is implemented exclusively for 2D images. It determines a global orientation estimation based on a principal component analysis (PCA) of the Fourier spectrum. This approach ensures high speed and robustness for the cases, in which the fibers are not well preserved and cannot be separated from each other, for example SEM micrographs of polycaprolactone PCL fibers [24].

The second method is mainly applied for 3D datasets with spatially separable fibers. It calculates the second-order structure tensor and derives its eigenvalues and eigenvectors, corresponding to the fiber orientation. This approach ensures better accuracy than the Fourier-based method but requires much more computational time, because the orientation is calculated for each fiber separately.

#### Fiber diameter estimation

The estimation of fiber diameters is provided by a function estimate_diameter_single_run. It requires a binary dataset obtained by segmentation and including orientation information at every point of the medial axis of the fibers, as obtained by the fiber orientation analysis. The method relies on emitting rays through the volume in directions perpendicular to the direction of the fiber at every point of the medial axis (Fig 4b). As a result, each of the rays gives two intersection points with the profile of the fiber section. Then, the distances between these points are calculated and averaged for all rays determining the fiber diameter at the respective coordinate of the volume.

#### Porosity estimation

The porosity calculation is provided by the function calc_porosity. The required input is a segmented dataset, which is composed of an arbitrary number of materials (M), labeled from 1 to M on a zero-background. The porosity is calculated as the fraction of the air volume or material volume to the whole volume [68,69].

#### Object counting

Objects can be counted by the object_counter function. It allows for counting and the estimation of various morphometric characteristics of non-adjacent particles and inclusions presented in the segmented dataset. Behind the scene, it performs a connected component analysis to assign different labels to the non-adjacent clusters of the voxels [70,71]. Then, characteristics are estimated for each separated object, and the calculation results are saved as a CSV file.

### Visualization

Visualization is a routine task for data analysis. In many cases it requires exporting data to the format required by the visualization software and needs adjusting many parameters. To avoid this extensive work, we added a module for visualization. It allows to visualize all extracted quantitative characteristics like orientation and diameter via histograms, heatmaps, and volume rendering to reveal so far hidden properties. The module is largely based on available packages, such as Matplotlib, VisVis, Mayavi2, and ipyvolume. If preprocessing is needed before visualization, a set of functions provided in the utils module described in the following simplifies this procedure.

### Utils

The volumetric visualization of fiber orientation requires mapping of geo-coordinates to a specific color. The function geo2rgb juxtaposes a color from the HSV color model to a pair of azimuth and elevation values.

#### Statistical information

For statistical analysis, such as the calculation of p-values, we included the function calculate_tukey_posthoc. To estimate p-values for several sample characteristics, the user passes an associative array with ’values’ and their ’type’, which store the array of sample characteristic values (for example porosity) and the array of labels (types of biomaterials) associating each value with a specific a group of samples. The result of the p-value calculation can be saved to a CSV file.

### Availability and Installation

The source code of quanfima is available from https://github.com/rshkarin/quanfima, conveniently packaged with a user manual, validation datasets and several examples demonstrating usage. Also, quanfima is available for download from the Python Package Index. The complete source code used in this paper is provided in the attached Jupyter notebook along with the real datasets for further plug and play use.

## Validation

To test the performance of the developed package, we use a set of generated datasets as well as several types of biomaterials. The cases selected here represent various types of widespread composite 3D scaffolds made from different biomaterials such as polymers, calcium phosphate ceramics, and bioglass at distinct levels of characterization, such as after production and *in vitro* analysis. While all of the data was acquired with micro-CT, the package can be equally applied to any imaging technique.

### Orientation evaluation

The orientation of fibers in 3D scaffolds is a key parameter, which determines some of the mechanical properties and the growth direction of biological tissues during the implantation process. Fibrous scaffolds are intended to be used in the regeneration of different tissues (bones, nerves, vessels, etc.) as they serve as constructions which mimic the topography of natural ECM of native tissues [6]. As an example, for the demonstration of the orientation analysis functionality of the developed module, we used 3D scaffolds made from PCL polymer with randomly oriented and aligned fiber structures.

**Fig 5:**
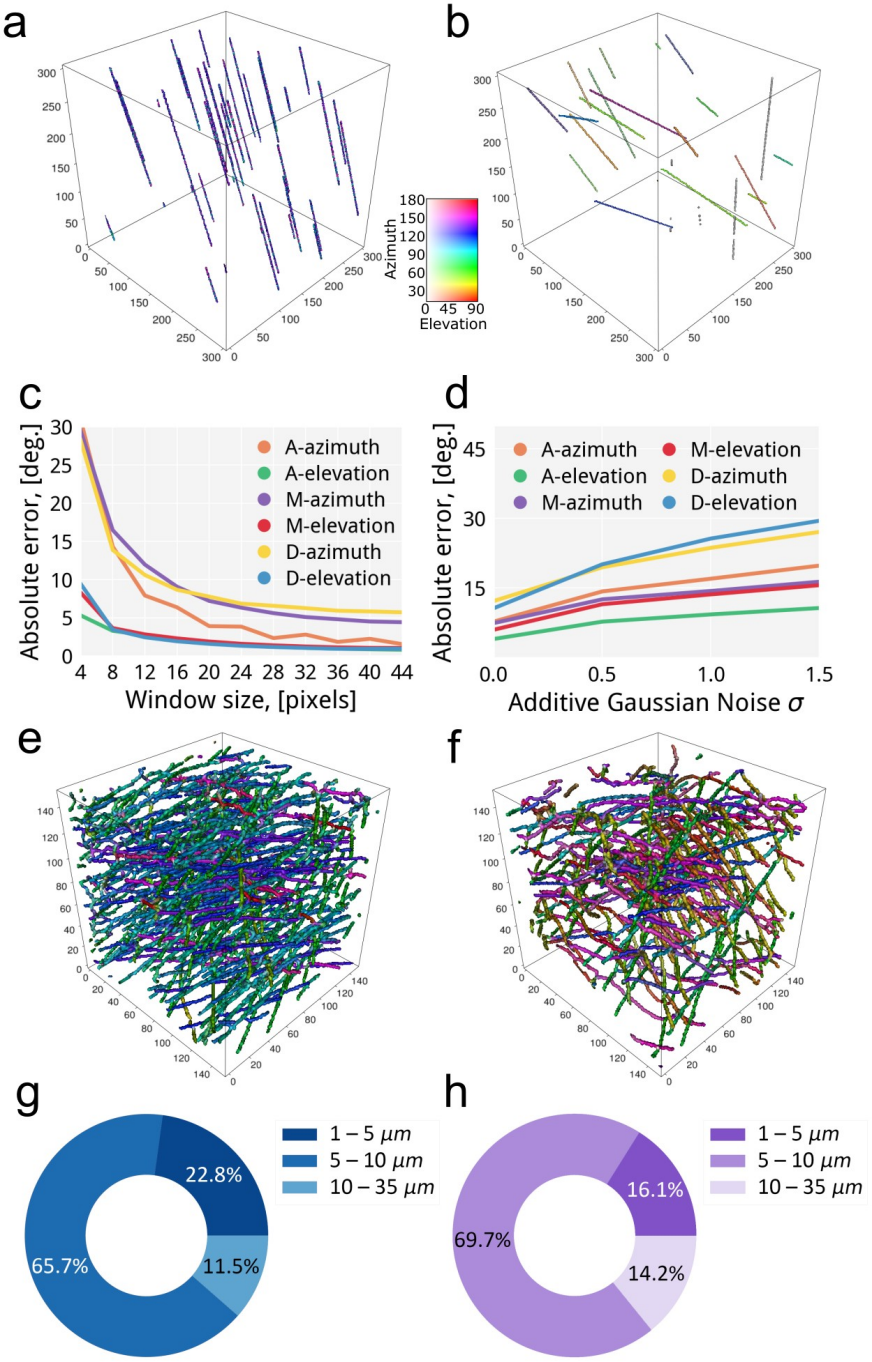
The accuracy analysis of the orientation algorithm: a,b) the visualization of the orientation estimation of the generated datasets with aligned and disordered configuration for 32 pixels of window size (the generated dataset is shown in Fig 1(a)–1(c)); c) the absolute error of orientation quantification using the implemented algorithm on the generated datasets without noise to determine the optimal window size; d) the dependency of absolute angular error derived from the results produced by the implemented algorithm on the generated datasets contaminated with different noise levels; e,f) the visualization of the orientation estimation of the wPCL and rPCL scaffolds; g,h) the measurements of fiber diameter distribution within scaffolds with well-aligned and randomly oriented structures.

First, we simulated different fiber configurations as a phantom sample, see Fig 1(a)–1(c). As the dataset is 3D, we estimated the orientation by a tensor-based method with a 3D local window of the specified size, see Fig 5(a) and 5(b).

Selection of an optimal window size was performed by estimating the orientation of fibers in the generated datasets without noise for a range of window sizes (from 4 to 44 pixels). The absolute error has been calculated as a sum of errors over the volume at the points where the tensor has been estimated. The azimuth error falls rapidly from 4 to 20 pixels of window sizes, and then weakly decreases further. The error is barely changing from the optimal window size of 32 pixels, see Fig 5c. This trend is persisted for different configurations of fibers. With this optimal window size of 32 pixels, the absolute errors of the datasets for azimuth and elevation are (2.79±2.11, 1.06±0.89), (5.09±14.62, 1.23±0.66) and (6.25±24.31, 1.05±0.66) degrees for aligned, moderately aligned and disordered fiber compositions correspondingly. This behavior is similar for both azimuth and elevation angular errors. Therefore, this window size was chosen for further data evaluation.

We also checked whether there are angular ranges where the algorithm loses accuracy. We have measured the angular error from -90 to 90 degrees for azimuth and from 0 to 90 degrees for elevation with a step of 5 degrees. The calculations, shown in S2(a)–S2(c) Fig**,** show that the maximum error increases with broadening of angular ranges. In other words, for more disoriented structures the error of orientation analysis increases. On the one side, this is due to fibers, which are oriented almost in one plane (close to the limits of azimuth and elevation) and a 2D analysis might be more accurate for such fibers. On the other side, tightly packed fibers might be difficult to resolve with the window size close to the diameter of the fibers.

The orientation error on real datasets might be large due to the noise. Therefore, we run the algorithm for each generated dataset with increasing noise level, σ_agn_ of 0.0, 0.5, 1.0, and 1.5. As it can be seen in Fig 5f, the errors slowly increase from σ_agn_ = 0.0 to 1.0 by 6.9 and 9.2 degrees for the aligned and moderately aligned fibers, respectively, while the disordered configuration has a steeper change of 11.5 degrees. Satisfactory algorithm accuracy from 0.0 to 1.0 of σ_agn_ is achieved for the aligned and moderately aligned configurations, whereas for the disordered configuration the satisfactory range is between 0.0 and 0.5 of σ_agn_. While this evaluation was done on small generated dataset, the package can be efficiently parallelized to up to 30 threads, S2d Fig. The throughput of the method part was estimated without considering any input/output (I/O) operations. The evaluation was done for each parallelization scenario of 1, 10, 20, and 30 threads for 3D data sets with side length of 256, 512 and 1024 pixels. The efficiency of parallelization almost linearly increases with the data size. However, within a single dataset, the throughput scales non-linearly. 10 parallel threads process the dataset almost 10 times faster than 1 thread. However, further doubling and tripling the number of threads resulted in a smaller performance improvement for 4 and 6 dataset scaling factors correspondingly. This loss in scalability is due to the overhead of thread creation, management, and memory transfers. Nonetheless, the parallelization of the package is a prerequisite for large datasets and faster analysis.

As the orientation analysis on the generated dataset showed to be robust in terms of window size and orientation error, we applied the same module for the orientation analysis of fibrous PCL scaffolds. The fabrication process of these scaffolds (electrospinning) allows to control the orientation of the fibrous and results in complex 3D structure. We used the morphology module of quanfima together with the integrated visualization module.

The 3D orientation analysis presented in Fig 5(d) and 5(e) ensures an immediate effortless visual evaluation of the scaffold structure depending on the fiber orientation. In the case of the aligned fiber structure, it can be clearly seen that most of the fibers are colored in green and blue colors, which means, that the preferential azimuth angle in concentrated in the range from 60 to 120 degrees. Some of the fibers in the well-aligned scaffolds clearly have different in-plane orientation. That might be the result of the fabrication process as each fiber layer is deposited individually. In the elevation, most of the fibers lie in the same plane, which is defined by the rotation speed of the collector during the electrospinning process. The PCL scaffold fabricated with randomly oriented fibers, clearly shows a large variability in fiber angles. In fact, some fibers seem to be linked in depth from one to another layer. Detailed analysis of fiber orientation with quanfima confirms the feasibility of the electrospinning technique to fabricate controlled structures with the defined fiber orientation [55].

### Fiber diameter

The fiber diameter plays a crucial role in the cell-scaffold surface interactions and allows to readily manipulate cellular activity during implantation [72]. Cell attachment and proliferation depends on whether fibers are of micro- and/or nanoscale [5].

As with fiber orientation, we evaluated the algorithm in terms of accuracy and performance on the generated datasets with a varied number of fibers. The results in S3 Fig a demonstrate that the accuracy of the algorithm is invariant to the number of fibers in the volume. The throughput (S3b Fig) varies much depending on structure of the fibers and ranges from 1.2×10^5^ to 6.1×10^5^ MB/s for the aligned, from 1.6×10^5^ to 5.2×10^5^ MB/s for the moderately aligned and from 4.0×10^5^ to 1.12×10^6^ MB/s for the disordered configuration. The number of skeleton voxels and the fiber diameter presented in the dataset influence the throughput, because a thicker fiber requires more iterations to detect a border. Thus, the algorithm assures the accuracy within a single pixel and performance completely depends on the number and the actual diameter of the fibers in the dataset.

The fiber diameter analysis was performed on pure polycaprolactone scaffolds with randomly oriented and well-aligned structures. According to the Fig 5(g) and 5(h), the influence of the collector rotation speed on the fiber diameter within scaffolds can be clearly seen. The comparison of the results obtained for randomly oriented and well-aligned structures demonstrated decrease of the fibers with the diameter in the range from 1 to 5 μm for 6.7%, from 5–10 μm for 4% and increase from 10–15 μm for 2.7%, respectively. The obtained results were expected, because with increasing the speed of the collector during electrospinning process, the stretching of the fibers occurred [55]. This phenomenon was correctly detected by the algorithm.

**Fig 6:**
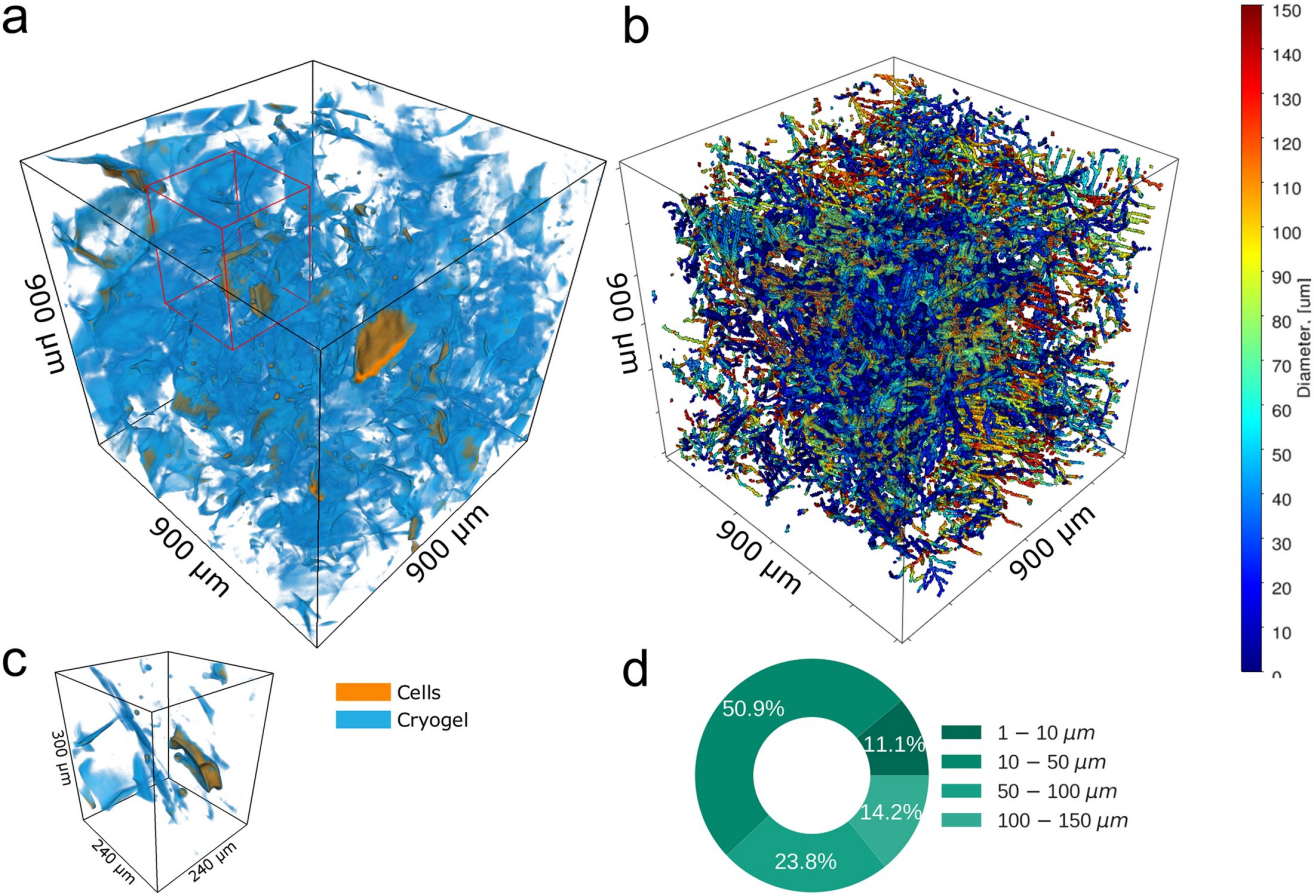
The 3D visualization of cryogel scaffolds with pancreatic cells (a) and diameter estimation of the scaffolds walls (b). The ROI enclosing a single pancreatic cell (c) and the pie chart (d) representing the amount of thin and thick scaffold walls.

Further, we investigated the diameter of the structures in cryogel scaffolds used for 3D cell culturing (Fig 6(a) and 6(b)) [61]. X-ray phase contrast tomography was used to localize pancreatic cells in 3D scaffold of cryogel after 21 days of cultivation, see Fig 6(a) and 6(c). The wall thickness of the cryogel scaffold was calculated with the fiber diameter module of quanfima. The characteristic geometry of the cryogels is demonstrated by a 3D view of fiber diameter (Fig 6b). The cryogel compromises two main substructures: thick walls laying parallel to each other with thin walls linking these layers in between. The wall thickness was found to be 44–85 μm for thick structures and 22–51μm for thin (Fig 6d). Such an architecture with thick walls in perspective may ensures good stiffness and mechanical strength of the cryogel scaffold. The volume with thin walls may lead to an effective uptake of cell media and provides smaller niches for cell adhesion.

### Porosity evaluation

The main properties of porosity, such as pore size and interconnectivity have a direct implication on the functionality of scaffolds during application. A porous structure with interconnected pores is essential for proliferation and migration of cells for tissue vascularization and formation of new tissues as well as delivery of nutrition and removal of a metabolic waste. The porosity also defines the mechanical properties and strength of the scaffold and thus is one of the key parameters in characterization of biomaterials.

**Fig 7:**
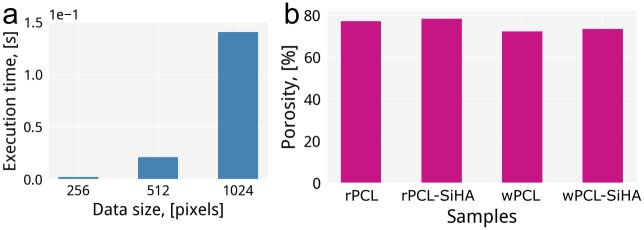
The performance evaluation of the porosity estimation approach over the scaled generated datasets (a) and the result of porosity estimation for the PCL scaffolds with and without inclusions (b).

We measured the performance of the algorithm for porosity calculation based on a generated dataset of a disordered configuration containing 100 fibers. The results obtained at different scales of the original volume demonstrate a non-linear scalability (Fig 7a), i.e. the increase of data size by a factor of two leads to an increase of evaluation time by a factor of 9.7.

Using functionality for porosity estimation in quanfima, we measured the porosity for pure and composite (containing SiHA microparticles) PCL scaffolds with well-aligned or randomly oriented fiber structures, see Fig 7b. For both fiber orientation cases, the enriched with SiHA scaffolds demonstrate slightly higher porosity values with the increase from 72.3% to 73.5% for well-aligned and from 77.2% to 78.4% for randomly oriented biomaterials. This increase in porosity might presumably positively influence cell penetration [55].

### Object counting evaluation

In the field of TE, the development of heterogeneous, composite scaffolds with different inclusions, such as calcium phosphate or bioglass particles is widespread. These inclusions determine different properties of a scaffold such us biocompatibility and chemical and mechanical properties. For this reason, a complete understanding of the amount of these inclusions in the scaffold structure based on the desired performance features is crucial for further development of biomaterials.

To evaluate the performance of the object counter, we have generated several datasets filled with 500 to 2500 non-intersected spheres of fixed size. From the results presented in S1b Fig**,** we find that the execution time increases almost linearly.

**Fig 8:**
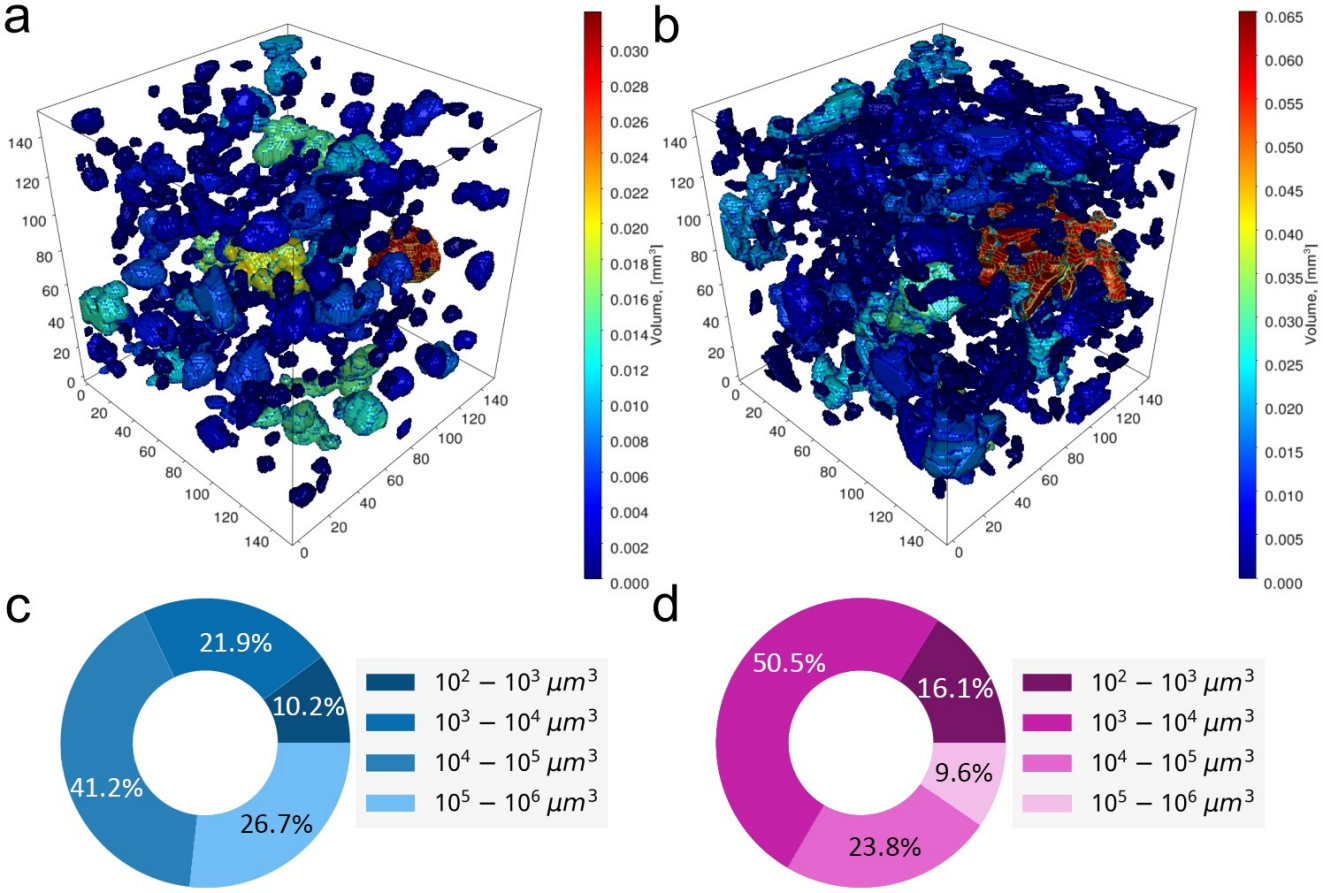
The analysis of hydrogels with BGSr (a) and α-TCP (b) particles and the measurements of particles distributions for each sample (c, d).

We analyzed mineralized hydrogels with two different types of inclusions: BGSr (Fig 8a) and α-TCP (Fig 8b). To maintain the injectability of the hydrogels, the particles included for mineralization should not agglomerate [58,59]. Analyzed particles are color-coded with respect to their volume, which allows for qualitative comparison of the particles with each other (Fig 8(a) and 8(b)). Further quantitative analyses over the whole sample volumes are shown in the pie charts (Fig 8(c) and 8(d)). We find that α-TCP particles agglomerate twice less as BGSr, which maintains the injectability of the hydrogels.

## Conclusions

We have presented the quanfima package for a complete, comprehensive 2D and 3D analysis of fibrous biomaterials. It provides capabilities for morphological analysis of fibrous structures and offers statistical analysis and visualization without bindings to a specific visualization system. The package can be easily applied to other biomaterials for characterization of wall thicknesses, inclusions, and porosity. The power of such analyses was demonstrated on various biomaterials, such as pure and composite PCL scaffolds, hydrogels and cryogels. In all cases, it provides quantitative 3D analysis and effortless visualization of the results. These examples are supplemented with the Jupyter notebook, making further use of the quanfima package possible as plug and play analysis for any datasets.

In future work, we will extend the functionality of the quanfima package by implementing new algorithms to analyze fiber intersection types, fiber clustering, and 3D shape. We will also include a new module for fiber tracking to obtain the information of individual fiber lengths. We also plan to improve the synthetic fiber generation algorithm which will no more be limited to straight fibers but allows for curved fibers with inclusions resembling particles and cells on the surface of the fibers. As quanfima package relies on a set of labels (segmentation) easily available, we will link the package with the available segmentation packages.

## Supporting information

S1 Fig The performance evaluation results of the generation of synthetic fibers and the object counting: a) the generation time of the datasets with different number of fibers in scenarios with and without fiber intersections; b) the duration of object counting for different number of particles with the constant size of the dataset.
(TIF)

S2 Fig The error-prone zones and the throughput evaluation of the implemented tensor-based method: a,b,c) the estimation of absolute error within the angular ranges to identify error-prone angular zones; d) the throughput evaluation of the implemented algorithm over the different sizes of the dataset for different scenarios of parallelization.
(TIF)

S3 Fig The accuracy (a) and performance (b) evaluation for the algorithm of diameter estimation.
(TIF)

S1 File The Jupyter notebook with the example of using the developed package. (IPYNB)
