# Supplementary figures and images for: Quanfima: An open source Python package for automated fiber analysis of biomaterials
Source: PLoS One. 2019 Apr 11;14(4):e0215137. doi: 10.1371/journal.pone.0215137 (PMC6459545; doi:10.1371/journal.pone.0215137)

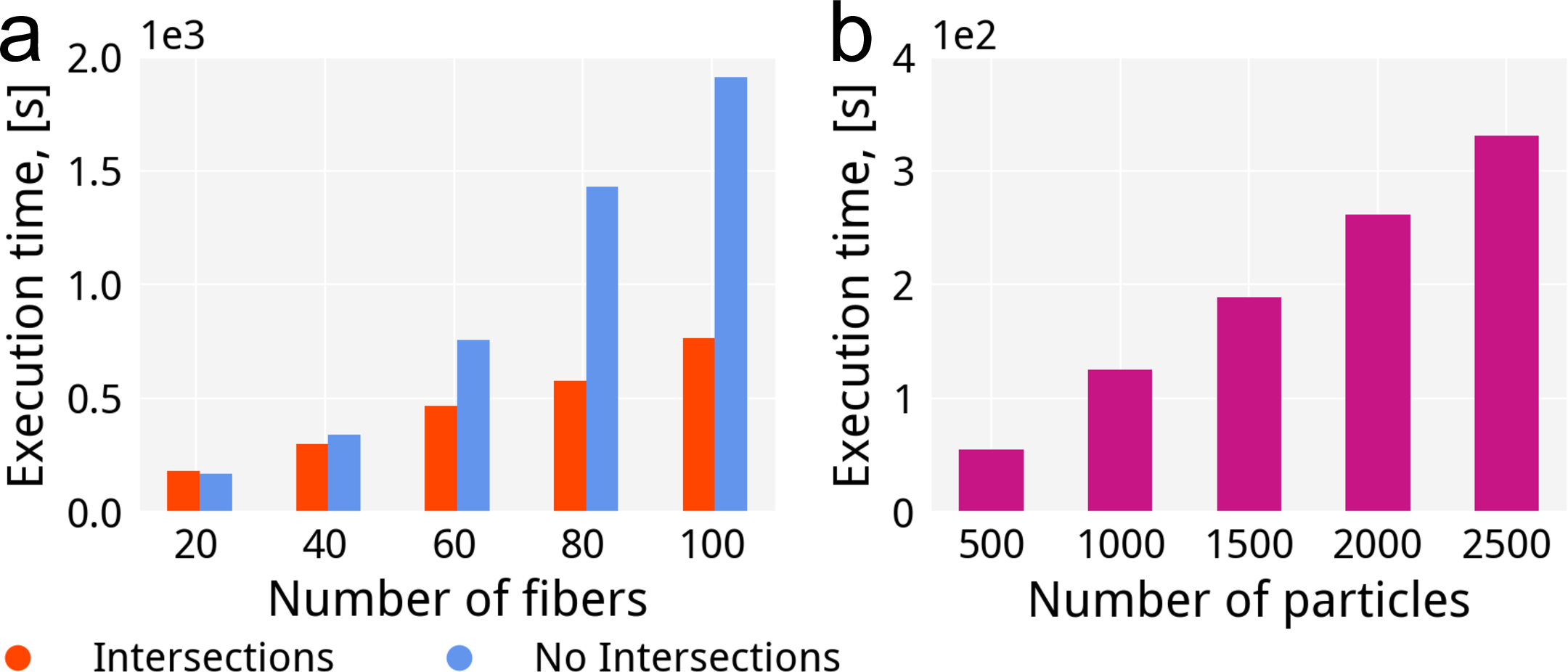

Supplement: S1 Fig — The performance evaluation results of the generation of synthetic fibers and the object counting: a) the generation time of the datasets with different number of fibers in scenarios with and without fiber intersections; b) the duration of object counting for different number of particles with the constant size of the dataset. (TIF) [file pone.0215137.s001.tif]

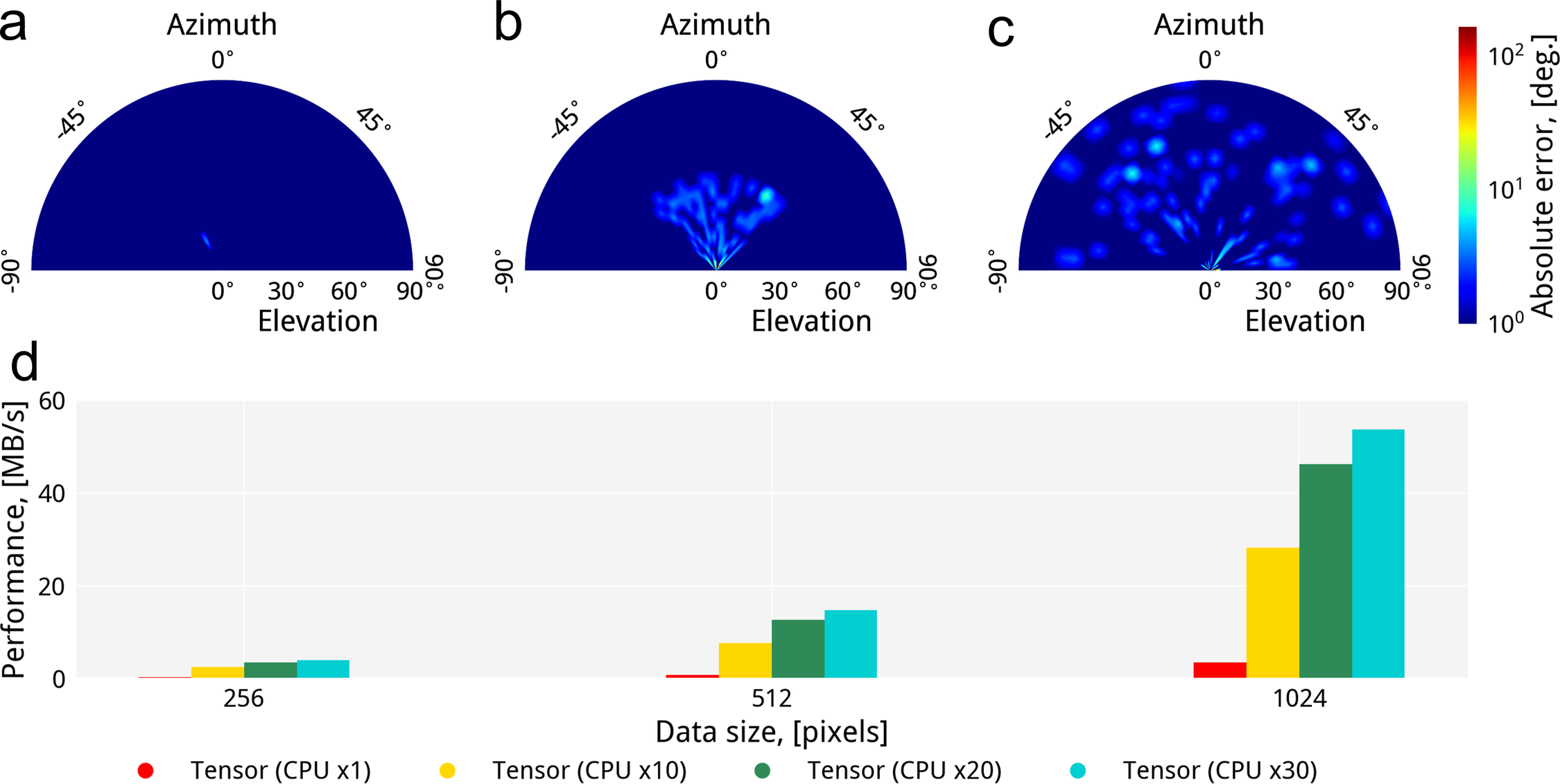

Supplement: S2 Fig — The error-prone zones and the throughput evaluation of the implemented tensor-based method: a,b,c) the estimation of absolute error within the angular ranges to identify error-prone angular zones; d) the throughput evaluation of the implemented algorithm over the different sizes of the dataset for different scenarios of parallelization. (TIF) [file pone.0215137.s002.tif]

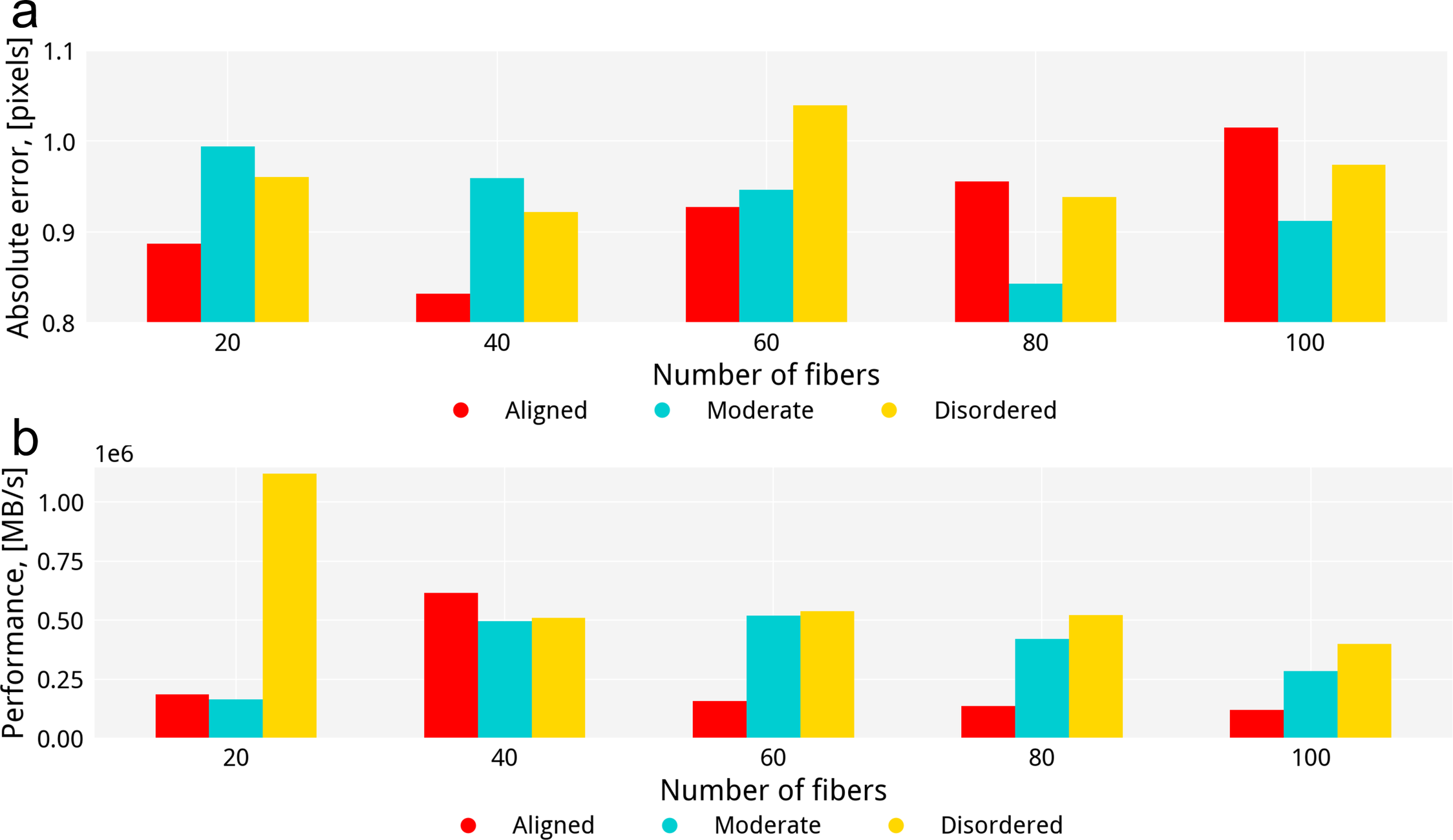

Supplement: S3 Fig — The accuracy (a) and performance (b) evaluation for the algorithm of diameter estimation. (TIF) [file pone.0215137.s003.tif]
